# Primary splenic lymphoma discovered on massive splenomegaly: A case report

**DOI:** 10.1016/j.ijscr.2022.107124

**Published:** 2022-04-27

**Authors:** Hazem Beji, Mahdi Bouassida, Ghazi Laamiri, Emna Chelbi, Salwa Nechi, Hassen Touinsi

**Affiliations:** a Department of General Surgery, Hospital Mohamed Taher Maamouri, Nabeul, Tunisia; b University Tunis El Manar, Faculty of Medicine of Tunis, Tunisia; c Department of Pathology, Hospital Mohamed Taher Maamouri, Nabeul, Tunisia

**Keywords:** Massive splenomegaly, Large B cell lymphoma, Splenectomy, Case report

## Abstract

**Introduction and importance:**

Malignant lymphoma occurs in all the systemic organs**.** Rarely, large B-cell lymphoma is located in the spleen, making the diagnosis difficult.

Herein, we report a patient presenting with massive splenomegaly due to LBCL. Splenectomy was essential to assess the diagnosis and to guide postoperative therapeutics.

**Presentation of a case:**

A 47-year-old woman, with no comorbidities, complained of weight loss and abdominal pain. She had a palpable spleen that extended below the navel.

CT scan revealed massive splenomegaly and lymph nodes in the spleen hilum. Splenectomy was performed.

Histopathological examination confirmed the diagnosis of large B-cell lymphoma.

The postoperative course was uneventful. Three courses of chemotherapy were given. The patient was in remission after a follow-up of 8 months.

**Discussion:**

Massive splenomegaly can be one of the circumstances of the discovery of large B-cell lymphoma.

Splenectomy was then essential to confirm the diagnosis and to guide postoperative therapeutics. It also permits reducing hypersplenism and preventing spleen rupture.

In patients with high operative risk, splenic needle biopsy should be taken into consideration.

Splenic artery embolization before surgery can also be performed in patients having massive splenomegaly to reduce the spleen volume.

We highlight the importance of splenectomy to confirm the diagnosis and to relieve the symptoms. Postoperative chemotherapy is essential to prevent relapses.

**Conclusion:**

Splenectomy is essential in spleen localized large B-cell lymphoma. It permits to confirm the diagnosis, relieve symptoms, and treatment of underlying hematologic malignancies. Postoperative chemotherapy is essential to prevent relapses.

## Highlights

• Spleen localized large B-cell lymphoma is a rare condition.
• Massive splenomegaly is rarely a circumstance of discovery of the disease.
• Splenectomy is essential to confirm the diagnosis and relieve symptoms.

## 1 Introduction and importance

Malignant lymphoma occurs in all the systemic organs. In comparison with Hodgkin Lymphoma, Non-Hodgkin's Lymphoma (NHL) has a greater predilection to touch extranodal sites, including the spleen [1], [2]. Rarely, large B-cell lymphoma is located only in the spleen, making the diagnosis difficult. Primary splenic lymphoma is a very rare entity. It is a disease confined to the spleen or at the most involves hilar lymph nodes with no recurrence after splenectomy [3], [4]. The place of splenectomy is still debated [5]. It is mainly important in providing histopathological confirmation, relieving the symptoms, and reducing hypersplenism.

Herein, we report a patient presenting with massive splenomegaly due to spleen localized LBCL. Splenectomy was essential to assess the diagnosis. This work has been reported in line with the SCARE 2020 criteria [6].

## 2 Presentation of a case

A 47-year-old woman, with no comorbidities, complained of weight loss and abdominal pain.

On the physical examination, she had no palpable peripheral lymphadenopathy. She had a palpable spleen that extended below the navel. There were no ascites or hepatomegaly.

Laboratory studies revealed haemoglobin of 9.3 g/dl, platelet count of 75,000/μl, white blood cells count of 5300/μl. Renal and hepatic functions were normal. LDH, uric acid, and serum Ca levels were normal.

**Fig. 1:**
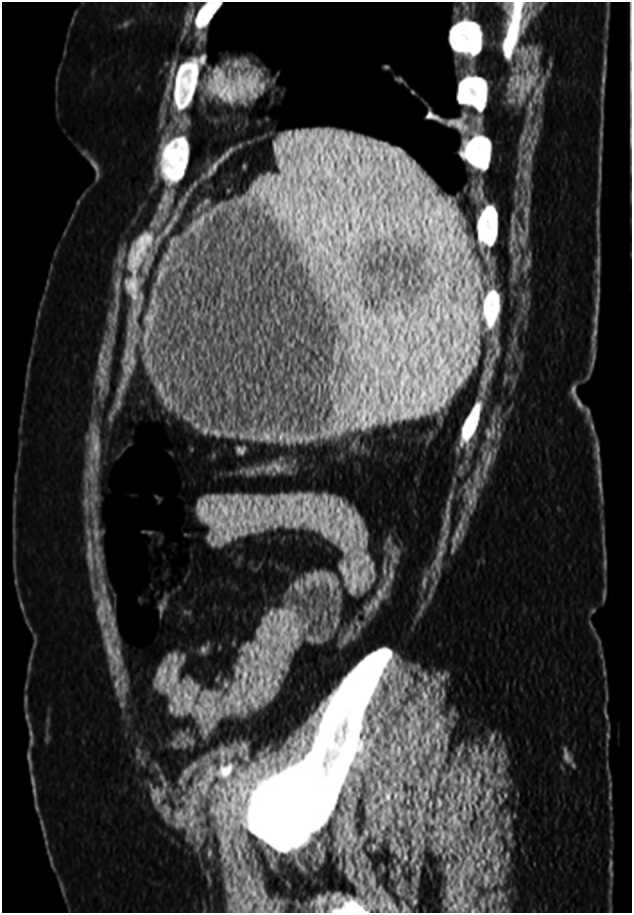
Sagittal CT scan showing massive splenomegaly and splenic hypodense lesions.

**Fig. 2:**
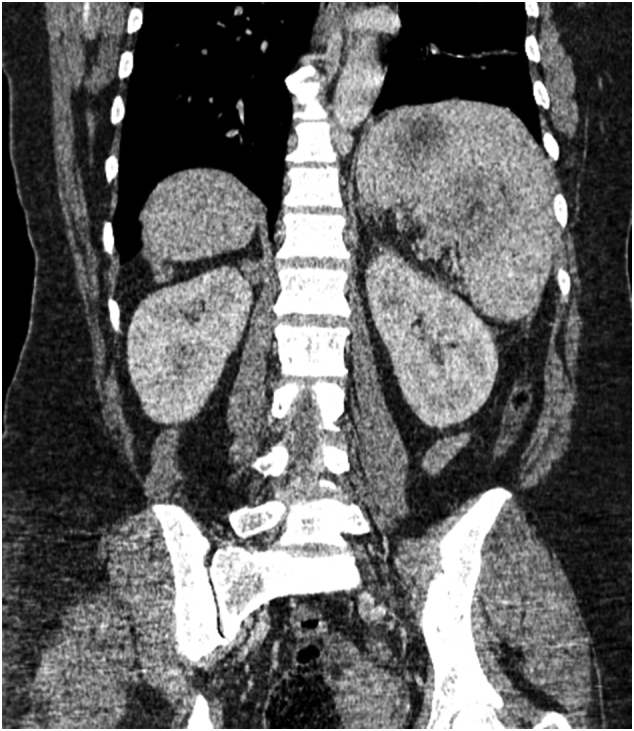
Coronal CT scan showing massive splenomegaly and lymph nodes in the splenic hilum.

CT scan revealed massive splenomegaly and lymph nodes in the spleen hilum (Fig. 1, Fig. 2). Splenectomy was planned.

The two essential diagnoses evoked were: lymphoma and abdominal tuberculosis.

We opted for laparotomy instead of laparoscopy because of the massive splenomegaly. A left subcostal incision was made. Intraoperative findings revealed lymph nodes in the splenic hilum. We performed splenectomy. It was performed by a surgeon with seven-year experience. The blood loss was estimated at 150 mL and the operation time was 150 min.

The resected spleen weighed 3.5 kg and measured 22 × 20 × 10 cm.

**Fig. 3:**
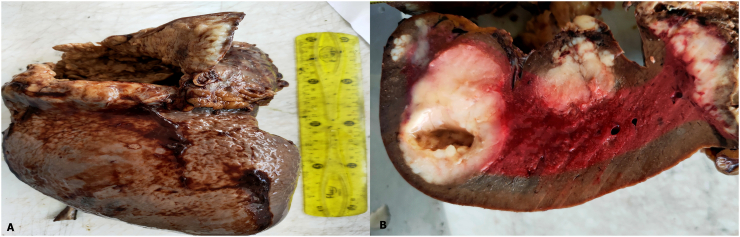
The resection specimen (A). The cut surface of the resected spleen containing grey-white tumors (B).

In the cut surface, there were grey-white homogenous tumors soft in consistency (Fig. 3).

**Fig. 4:**
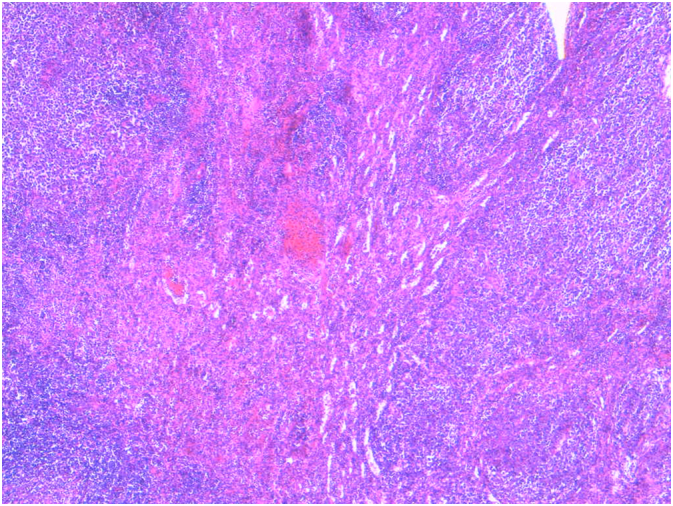
Microscopy showing the proliferation of large neoplastic lymphoid cells.

**Fig. 5:**
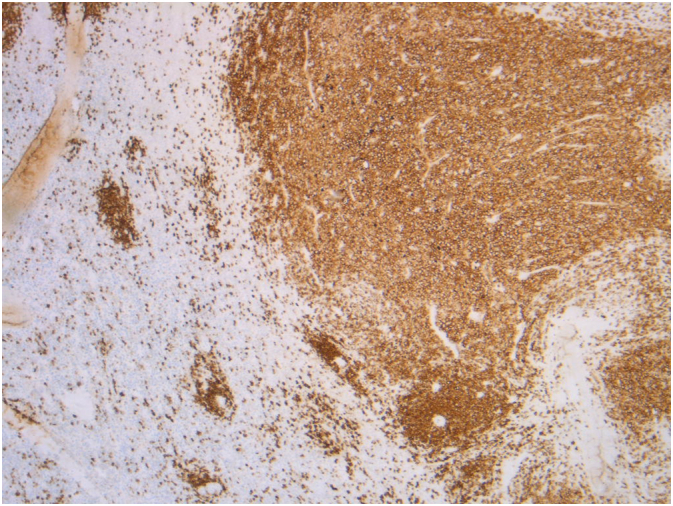
Immunohistochemistry positive for CD 20.

Microscopy showed the proliferation of large neoplastic lymphoid cells (Fig. 4). Immunohistochemical staining confirmed the diagnosis of large B-cell lymphoma. The tumor cells were immunopositive for CD 20 (Fig. 5).

The disease was classified low risk according to the international prognostic index for non-Hodgkin lymphoma [7].

The patient received antibiotic prophylaxis with Penicillin 1000 mg/12 h. This treatment will last two years.

The postoperative course was uneventful. Three courses of standard CHOP (Cyclophosphamide, Hydroxydaunorucibin, Oncovin, Prednison) plus rituximab chemotherapy were given. The patient was in remission after 8 months without any complications related to disease and splenectomy.

## 3 Clinical discussion

Here, we reported a case of a spleen localized LBCL in a patient presenting massive splenomegaly and hypersplenism. Splenectomy was essential to relieve symptoms, confirm the diagnosis, and therefore guide postoperative therapeutics.

The main weakness of our work is the short follow-up period and the non-performance of a positron emission tomography (PET) scan which is important in the disease staging.

Large B-cell lymphoma is the most frequent non-Hodgkin's lymphoma representing 33% [8]. The diagnosis is difficult essentially when those tumors disseminate to extranodal sites such as the spleen.

If the rigorous criteria given by Das Gupta et al. are followed [3], primary lymphoma of the spleen is an extremely rare condition. The incidence is less than 1% of all NHL [5].

Splenomegaly and hypersplenism can be one of the circumstances of the discovery of this pathology. Computed tomography (CT) scan is not performant to confirm the diagnosis [9]. A splenic needle biopsy can be a good option to assess the diagnosis without severe complications [10]. In patients with high operative risk, this technique should be taken into consideration.

Weight loss, the presence of lymph nodes in the spleen hilum were suggestive of lymphoma in our case. But tuberculosis, which is endemic in Tunisia, couldn't be ruled out [11].

Laparoscopy has become the most common approach for elective splenectomy. Its use for massive splenomegaly is controversial. Some authors recommend the use of laparoscopy regardless of the spleen size [12]. But due to technical difficulties including limited working space, adhesions to surrounding structures, challenges with retraction and retrieval, and possible trauma to dilated veins or the splenic capsule with subsequent bleeding, laparotomy is the gold standard treatment according to the European Association for Endoscopic Surgery (EAES) [13].

Splenectomy was then essential to confirm the diagnosis and to guide postoperative therapeutics. It also permits reducing hypersplenism and preventing spleen rupture [1].

Splenic artery embolization before surgery can also be performed in patients having massive splenomegaly to reduce the spleen volume. It reduces bleeding and makes spleen manipulation simpler [14].

It was reported that splenectomy for massive splenomegaly has a high rate of perioperative mortality, nearing 20% [15], [16]. This could be due to the rapid progression of the disease [17].

Patients after splenectomy are at significant risk of infection by encapsulated bacteria such as *Streptococcus pneumoniae*, *Haemophilus influenzae* type B, and *Neisseria meningitidis*. Prophylactic antibiotics along with the adoption of appropriate vaccination are necessary to prevent post-splenectomy infections [18]. Postoperative anticoagulation therapy is mandatory to avoid portal or splenic vein thrombosis [9].

Most cases of aggressive lymphoma such as large B-cell lymphoma show fast disease expansion and progression, requiring immediate adjuvant chemotherapy [1].

In summary, we reported a case of spleen localized lymphoma in a patient presenting massive splenomegaly and hypersplenism. We highlight the importance of splenectomy to confirm the diagnosis, relieve the symptoms, reduce hypersplenism, and avoid splenic rupture.

## 4 Conclusion

Splenectomy is essential in spleen localized large B-cell lymphoma. It permits to confirm the diagnosis, relieve symptoms, and treatment of underlying hematologic malignancies. Postoperative chemotherapy is essential to prevent relapses.

## Sources of funding

This research did not receive any specific grant from funding agencies in the public, commercial, or not-for-profit sectors.

## Ethical approval

Not required.

## Patient consent

Written informed consent was obtained from the patient for publication of this case report and accompanying images. A copy of the written consent is available for review by the Editor-in-Chief of this journal on request.

## Guarantor

Dr. Beji Hazem

Dr. Mahdi Bouassida

## Provenance and peer review

Not commissioned, externally peer reviewed.

## CRediT authorship contribution statement

Hazem Beji and Mahdi Bouassida did the conception and design of the work, the data collection, and the data analysis and interpretation.

Ghazi Laamiri and Emna Chelbi did the critical revision of the article

## Declaration of competing interest

No conflicts of interest.
